# A call to action to address escalating global threats to academic research

**DOI:** 10.1016/j.xinn.2024.100758

**Published:** 2025-01-04

**Authors:** Gaelle Piret, Fun Man Fung, Josie Fullerton, Giuseppe Fico, Dmitriy Ponkratov, Wenxin Chen, Daniela Latorre, Kirsty Y. Wan, Nima Aghaeepour, Jules Welgryn, Adeel Razi, Patricia Silveyra, Ahmet Altun, Renata Z. Jurkowska, Alice C. Hughes, Joy Wolfram

**Affiliations:** 1 INSERM U1205, UGA, 38400 Saint Martin d'Hères, France; 2 School of Chemistry, University College Dublin, D04 C1P1 Belfield, Ireland; 3 UCD Geary Institute for Public Policy, University College Dublin, D04 N9Y1 Dublin, Ireland; 4 School of Cardiovascular & Metabolic Health, University of Glasgow, G12 8TA Glasgow, UK; 5 European Alliance for Medical and Biological Engineering and Science, 3001 Leuven, Belgium; 6 Life Supporting Technologies, Universidad Politécnica de Madrid, 28040 Madrid, Spain; 7 Siemens Digital Industries Software, B90 8BG Solihull, UK; 8 Department of Philosophy of Science and Technology, University of Science and Technology of China, Hefei 230026, China; 9 Institute of Microbiology, ETH Zurich, 8093 Zurich, Switzerland; 10 Living Systems Institute & Department of Mathematics and Statistics, University of Exeter, EX4 4QD Exeter, UK; 11 Department of Anesthesiology, Pain, and Perioperative Medicine, Department of Pediatrics, Department of Biomedical Data Science, Stanford University School of Medicine, Stanford, CA 94305, USA; 12 Université Paris Nanterre, 92000 Nanterre, France; 13 Turner Institute for Brain and Mental Health, School of Psychological Sciences, and Monash Biomedical Imaging, Monash University, Clayton, VIC 3800, Australia; 14 Wellcome Centre for Human Neuroimaging, University College London, WC1N 3AR London, UK; 15 CIFAR Azrieli Global Scholars Program, Toronto, ON M5G 1M1, Canada; 16 Department of Environmental and Occupational Health, Indiana University School of Public Health Bloomington, Bloomington, IN 47408, USA; 17 Department of Pharmacology, Sivas Cumhuriyet University, Sivas 58140, Turkey; 18 Division of Biomedicine, School of Biosciences, Cardiff University, CF10 3AX Cardiff, UK; 19 School of Biological Sciences, University of Hong Kong, Hong Kong SAR 999077, China; 20 Australian Institute for Bioengineering and Nanotechnology, The University of Queensland, Brisbane, QLD 4072, Australia; 21 School of Chemical Engineering, The University of Queensland, Brisbane, QLD 4072, Australia

## Abstract

This article is a call to action to address escalating threats to scientific progress that affect academic researchers across the globe. These threats include public mistrust of science, challenges in translating academic research to end-user applications, a disconnect between academics and policymakers, emerging barriers to international collaboration, and a reliance on conventional metrics to evaluate academic performance. This article presents various calls to action informed by exemplary approaches across the globe that serve as frameworks to drive beneficial transformation for researchers, academic institutions, and society.

## Main text

**Figure 1:**
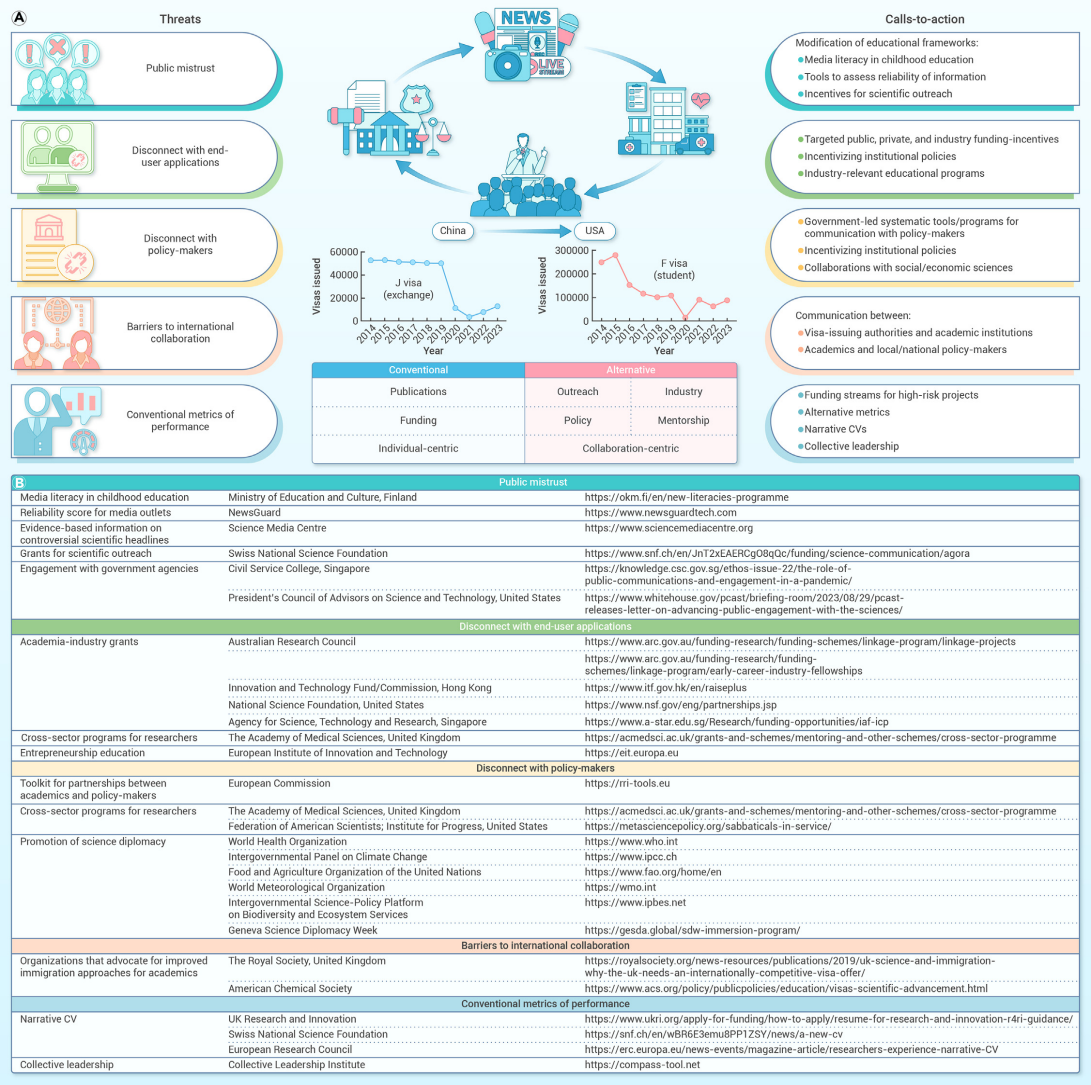
Calls to action to address escalating threats to scientific progress and impact in academia (A) Summary of escalating threats with examples of calls to action. United States visa data were obtained from the United States Department of State, Bureau of Consular Affairs (non-immigrant visas issued to citizens of Mainland China in each fiscal year; travel.state.gov). The J visa is common for academic researchers, but includes other categories unrelated to scientific research. (B) Examples of exemplary resources to address threats.

The World Laureates Forum is a global scientific meeting for prize-winners, including Nobel Prize laureates. The 2023 World Laureates Forum also brought together emerging leaders from across the world to discuss escalating threats to the progress and impact of academic research. This article outlines such intensifying threats with the purpose of providing collective calls for action to researchers, academic institutes, supportive organizations, and governments (Figure 1A).

## Public mistrust

Gaps in the educational system to prepare individuals to navigate misinformation are fueling the lack of public trust in science. Scientific literacy and trust in science among the public are critical for sustaining academic research funding and implementing government policies that support research progress and impact.

Addressing the global decline in public trust in science requires urgent changes to educational frameworks. This call to action advocates for curricula that teach students to critically evaluate scientific information, with a focus on integrating media literacy. Finland leads the way by incorporating media literacy across various school subjects.^1^ In language and literature, students analyze texts from various sources to contextualize and evaluate the reliability of information. In social studies, students critically analyze how current events are portrayed in the media. Media literacy can also be improved by making students aware of resources that provide evidence-based information on controversial scientific headlines or reliability scores for media outlets (Figure 1B). Incentives should also be provided for scientific outreach initiatives to foster dialogue between researchers and lay audiences (Figure 1B). Governments play a key role in building trust with the public, and some government agencies have outlined key principles to foster engagement between federal research agencies, the public, and science experts (Figure 1B).

## Disconnect with end-user applications

Translating academic research into end-user applications presents several challenges, including a disconnect between universities and industry partners with the capacity for large-scale implementation and commercialization. In the biopharmaceutical space, the “valley of death” is a term coined to illustrate difficulties in taking discoveries from the laboratory bench to the patient bedside, a process that costs billions of dollars, necessitating industry collaborations. Successfully overcoming this disconnect results in new medicines emerging from academic laboratories.^2^ A disconnect often exists between academia, industry sectors, and end users, stemming from a lack of supportive organizational structures, minimal government buy-in, and limited cross-sector training of academics. A lack of professional incentives, reflected, for example, in performance indicators, makes it challenging for academics to pursue industry collaborations. Operational bottlenecks also include limited incentives for company partners, such as large overhead costs on industry-sponsored research.

The call to action is to boost academic-industry and end-user collaborations through the establishment of targeted public/private/industry funding, incentivizing university policies, and hubs within universities to facilitate entrepreneurial growth (Figure 1B). Additionally, there is a need to develop industry-relevant educational programs within academia, including entrepreneurship training and cross-sector studentships, fellowships, and secondments (Figure 1B).

## Disconnect with policymakers

Policy decisions rely on scientific evidence; however, a disconnect exists between academic researchers and policymakers. Systematic tools and resources for academics to interact with policymakers are largely lacking.^3^ A major funding source for academic research is from the taxpayer, whose support enables the generation of scientific knowledge, products, and services. However, without a streamlined process for incorporating such knowledge and innovation into public policy, the impact of taxpayer investments becomes limited. Members of national science academies and government advisory councils/committees serve as important spokespeople who promote bidirectional dialogue between researchers and policymakers. There has been a push for advisory organizations to develop and support inclusive mechanisms to broaden the intake of evidence-backed advice.^4^

The call to action urges governments to create tools that foster communication between researchers and policymakers, including programs to enhance scientific literacy for policymakers and policy literacy for scientists. Funding opportunities that support research-policy integration are essential. The European Commission’s Horizon 2020 program, with its “responsible research and innovation” approach and accompanying toolkit (RRI Toolkit), promotes researcher-government collaboration (Figure 1B). For example, tools like “living labs” facilitate the co-development of solutions and regulations through feedback from both researchers and policymakers.^5^ Opportunities that enable academics to gain experience in government posts are also valuable for bringing new perspectives to policymaking and research (Figure 1B). Additionally, researchers in the natural sciences are likely to develop improved strategies for addressing policies and policymakers by collaborating with researchers from social sciences.

The accelerating pace of technological advancement and the interconnectedness of global challenges highlight the importance of science diplomacy in advocating for collaborative, international solutions over unilateral national approaches. Several international platforms and organizations exist to facilitate effective communication and cooperation to address urgent threats to people and the planet (Figure 1B).

## Barriers to international collaboration

Rising geopolitical tensions have hindered international research collaborations, while the pandemic served as a major catalyst for reduced exchanges due to border closures. Post-pandemic, these exchanges are at their lowest in a decade. For example, academic exchanges between China and the United States have decreased substantially, as reflected in a sharp drop in student and research exchange visas (Figure 1A). Additionally, researchers from lower-income economies often face visa denial for scientific conferences, as authorities deem their financial status insufficient to guarantee their return.^6^ Other barriers to international collaboration include funding agencies that exclude certain countries from grants, such as the Wellcome Trust in the United Kingdom, which bars co-investigators from Mainland China. Political tensions have also caused delays in the United Kingdom’s participation in the European Commission’s Horizon Europe program, forcing new grantees to leave the United Kingdom.^7^

Fragmentation of science impedes innovation and undermines progress toward achieving goals that require concerted global efforts, such as effective management of food/energy security, climate change, biodiversity collapse, and infectious diseases. This call to action is to improve communication between visa-issuing authorities and academic institutions. Academics are encouraged to contact local and national policymakers to generate awareness of the impact of visa restrictions. In some cases, this may involve universities taking legal action to oppose new laws that restrict foreign scientific talent from entering the country and workforce.^8^ Other strategies include preparing open letters to oppose visa restrictions and raising general awareness of the societal contribution that foreign academics make. In the past two decades, 38% of American Nobel Prize laureates in physics, chemistry, and physiology or medicine were immigrants.^8^ Overcoming barriers to international research collaboration is also a central mission of several organizations (Figure 1B). Academics are encouraged to communicate with such organizations for concerted efforts to drive change.

## Conventional metrics of performance

A career as an academic researcher is increasingly reliant on maintaining publication and funding metrics. Academic researchers are forced to adhere to a short cycle of “discovery to output” to continuously meet performance indicators. Research projects that yield incremental results are performed, as such undertakings increase the likelihood of securing funding and achieving publication outputs in the short term. Long-term research projects with the potential for groundbreaking discoveries may remain unpursued, as such endeavors are unfavorable for meeting metrics in the short term, jeopardizing the immediate job security of academics. Lack of academic job security and professional incentives linked to conventional metrics may play a role in the recent increase of retracted scientific publications due to scientific misconduct, particularly in Saudi Arabia, Pakistan, Russia, and China.^9^ Among numerous repercussions, misconduct erodes trust, raising concerns for public resource allocation to science.

Furthermore, the current performance evaluation system values individual-centric metrics, such as lead investigator positions on grants and first/last authorship positions on publications, above collaborative efforts. Accordingly, researchers are incentivized to pursue personal advancement over collective scientific progress, fostering a competitive rather than collaborative research culture that undermines the very foundation of academic inquiry. High levels of competition also fuel an unkind and aggressive working environment, as perceived by 78% of researchers in a global survey by the Wellcome Trust, United Kingdom, in 2020. Additionally, a conventional metric-centric evaluation system makes it challenging to bridge gaps between university research, industry, the public, and policymakers due to a lack of unconventional performance indicators. Systems that prioritize conventional metrics exclude individuals with alternative career trajectories that bring much-needed cross-sector expertise.

The call to action is for the development of diversified targeted funding streams to allow the exploration of high-risk projects. Funding agencies and academic organizations are also encouraged to recognize alternative metrics in the evaluation of academic performance, including industry partnerships, collaboration, impact on policy, outreach activities, and mentorship. Examples of this type of practice include narrative CVs, such as those adopted by several funding organization (Figure 1B). Successful translation of academic research to societal benefits often requires diverse collective efforts. There is a need for performance evaluation systems that value collective leadership in academia. Collective leadership involves embracing multiple perspectives and interests with a focus on relationship management, enabling system-oriented cooperation within and beyond the academic setting. Educational programs have been developed to cultivate collective leadership, such as those in sustainable development, through co-creation between universities and regional actors.^10^

## Outlook

Addressing escalating threats to scientific progress and impact requires a concerted effort to bridge gaps in science education and communication, fostering an environment where scientific inquiry is embraced as a cornerstone of civic engagement. The escalating threats are exacerbated by the isolation of academic researchers from the public, the media, industry collaborators, international colleagues, policymakers, and university executives. It is critical for academia to build meaningful connections to enhance research progress and impact, which requires a shift toward more cross-disciplinary thinking and a greater recognition for multiple modes of scientific impact. Prioritization of diversity is critical to ensure that a wider range of perspectives and expertise are integrated into academic research, ultimately strengthening the bridge between science and society and leading to a more sustainable research environment. The reinvigoration of academia is dependent on the collective efforts of academics worldwide. Our colleagues are encouraged to join the mission of advocating for change to bring benefits to individual researchers, academic institutions, and society as a whole.

## Acknowledgments

The conference organizers (World Laureates Association) had no role in the conception, design, preparation, funding, or publication of the article but were made aware of the article prior to submission. The work in the authors' laboratories is funded by the 10.13039/501100000691Academy of Medical Sciences (UK) under award number SBF007\100176 (R.Z.J.), 10.13039/100014013UK Research and Innovation under award number MR/X032914/1 (R.Z.J.), and the 10.13039/501100001711Swiss National Science Foundation under award number PR00P3_185742 (D.L.). The content is solely the responsibility of the authors and does not necessarily represent the official views of the affiliated organizations or funding agencies.

## Declaration of interests

W.C. has an affiliation with the World Laureates Association but contributed to this article in the capacity of their primary academic affiliation.
